# Representativeness of Radiologically Guided Fine-Needle Aspiration Biopsy of Bone Lesions

**DOI:** 10.1080/1357714021000022159

**Published:** 2002-06

**Authors:** Veli Söderlund, Edneia Tani, Henryk Domanski, Andris Kreicbergs

**Affiliations:** 1 Department of Radiology Karolinska Hospital Stockholm S-171 76 Sweden; 2 Department of Pathology and Cytology Karolinska Hospital Stockholm S-171 76 Sweden; 3 Department of Orthopaedics Karolinska Hospital Stockholm S-171 76 Sweden; 4 Department of Pathology and Cytology Lund University Hospital Lund Sweden

## Abstract

The consistency of the cellular yield as obtained by radiologically guided fine-needle aspiration biopsy (FNAB) was investigated in 29 cases with bone lesions. Aspirates from three different sites of the same lesion were analysed randomly and independently by two cytologists unaware of the clinical and radiological findings.The series was grouped cytologically into four categories: (1) benign, (2) sarcoma, (3) other malignancy, (4) non-conclusive. A lesion was considered cytologically homogenous, when all three aspirates were identically categorised. Among 29 lesions, 13 and 12, respectively, were assessed as homogeneous by the two cytologists. In the remaining lesions, heterogeneity almost exclusively pertained to the mixture of conclusive and non-conclusive aspirates. An alternative diagnosis was suggested in one case by each cytologist. Comparison of the two cytologists' assessments showed that 21 cases were compliant, i.e., no inter-observer difference in 63 out of 87 aspirates. In the remaining eight cases (24 aspirates), non-compliance was mainly due to differences between the cytologists in the ratio of conclusive versus non-conclusive aspirates. Only the analysis of one and the same aspirate resulted in two different diagnoses. A correct diagnosis was given by the cytologists in 22 and 23 cases, incorrect in two and non-conclusive in five and four, respectively.

Our cytological study of bone lesions, albeit limited, suggests that true tumour heterogeneity is rare. The non-compliance between the two cytologists and the diagnostic difficulties should mainly be attributed to the blind, random approach of the study.The main problem of FNAB pertains to the high rate of non-conclusive aspirates.This, however, does not entail an increased risk of incorrect diagnosis, but rather prompts repeat FNAB.


*Sarcoma* (2002) **6**, 61–68

Taylor&Francis healthsciences

**ORIGINAL ARTICLE**

### Representativeness of radiologically guided fine-needle aspiration biopsy of bone lesions

VELI SÖDERLUND[1], EDNEIA TANI[2], HENRYK DOMANSKI[3] & ANDRIS KREICBERGS[4]

*[1]Department of Radiology [2],Department of Pathology and Cytology, and [3]Department of Orthopaedics, Karolinska Hospital, S-171 76 Stockholm, Sweden; [4]Department of Pathology and Cytology, Lund University Hospital, Lund, Sweden*

**Abstract**

The consistency of the cellular yield as obtained by radiologically guided fine-needle aspiration biopsy (FNAB) was investigated in 29 cases with bone lesions. Aspirates from three different sites of the same lesion were analysed randomly and independently by two cytologists unaware of the clinical and radiological findings. The series was grouped cytologically into four categories: (1) benign, (2) sarcoma, (3) other malignancy, (4) non-conclusive. A lesion was considered cytologically homogenous, when all three aspirates were identically categorised. Among 29 lesions, 13 and 12, respectively, were assessed as homogeneous by the two cytologists. In the remaining lesions, heterogeneity almost exclusively pertained to the mixture of conclusive and non-conclusive aspirates. An alternative diagnosis was suggested in one case by each cytologist. Comparison of the two cytologists' assessments showed that 21 cases were compliant, i.e., no inter-observer difference in 63 out of 87 aspirates. In the remaining eight cases (24 aspirates), non-compliance was mainly due to differences between the cytologists in the ratio of conclusive versus non-conclusive aspirates. Only the analysis of one and the same aspirate resulted in two different diagnoses. A correct diagnosis was given by the cytologists in 22 and 23 cases, incorrect in two and non-conclusive in five and four, respectively.

Our cytological study of bone lesions, albeit limited, suggests that true tumour heterogeneity is rare. The non-compliance between the two cytologists and the diagnostic difficulties should mainly be attributed to the blind, random approach of the study. The main problem of FNAB pertains to the high rate of non-conclusive aspirates. This, however, does not entail an increased risk of incorrect diagnosis, but rather prompts repeat FNAB.

**Key words:** *bone lesions, representativeness FNAB, cytology, reproducibility, heterogeneity*

### Introduction

Cytology is widely accepted in the diagnosis of certain tumour entities like mammary and thyroid neoplasia. However, in the diagnosis of mesenchymal and especially bone lesions fine needle aspiration biopsy (FNAB) has been met with scepticism, despite a number of reports supporting the utility of this procedure.[1–3] Thus, histopathological analysis of tissue material from open biopsy or core biopsy remains the gold standard in the diagnosis of bone lesions[4]. The main criticism against cytology in the diagnosis of bone tumours is based on the anticipated difficulties in obtaining representative cell material because of tumour heterogeneity.[5,6] FNAB would seem to entail an increased risk of erroneous diagnosis compared to open biopsy because of the limited cellular yield from one specific site within each lesion. Thus, the cell material cannot be considered to reflect the whole of a tumour, since the latter may harbour areas of varying tissue differentiation. So far, however, the problem of tumour heterogeneity in conjunction with FNAB has not been addressed.

We investigated the consistency of the cellular yield by analysing multiple aspirates from the same lesions as assessed individually by two cytologists previously not informed about the clinical and radiological features of the patients.

### Material and methods

### *Patients*

The series included 30 consecutive patients referred to the Department of Radiology in 1998 for fine

Correspondence to: Veli Söderlund, ADR, Karolinska Hospital, S–171 76 Stockholm, Sweden. Tel.: +46–8-5177–000; Fax: +46–8-5177–4583; E-mail: veli.soderlund@ks.se

ISSN 1357–714X print/ISSN 1369–1643 online/02/0200061–08 © Taylor & Francis Ltd
DOI: 10.1080/1357714021000022159

needle aspiration biopsy of a bone lesion. Mean age of the 13 females and 17 males was 49 (3–84) years. Seven patients had a history of prior malignancy; colon (one), lung (one) and breast (two) carcinoma, lymphoma (one), Ewing's sarcoma (one) and high-grade osteosarcoma (one). The remaining 23 patients had no clinical history of neoplasia or skeletal disease.

### *Diagnosis*

The treatment diagnosis of the patients was established by combining data on clinical history and features, radiological findings and cytology in 29 cases, whereas one case (GCT) consistently yielding non-conclusive cytology was diagnosed by histopathology (open biopsy). The cytological diagnosis was confirmed by histopathology of postoperative material in ten cases. Thirteen patients had a benign bone lesion: six benign bone tumours (four GCT, two enchondroma), one fibrous dysplasia, one gout, one degenerative bone cyst, two infections and two normal. Six patients had a sarcoma (three osteosarcomas, one chondrosarcoma, one malignant fibrous histiocytoma (MFH), one chordoma) and 10 other bone malignancies: cancer metastasis (four cases), myeloma (three cases) or lymphoma (three cases).

### *Radiography*

All patients, except one with a lesion of the bone marrow identified by MRI, had lytic lesions, although there was a variation in bone and periosteal response. Table 1 shows the distribution of the series according to site, growth pattern,[7] tentative radiological diagnosis and final diagnosis.

### *Fine needle aspiration biopsy*

The fine needle aspiration biopsy (FNAB), always involving a radiologist and a cytologist, was performed as an out-patient procedure. The lesions were identified by fluoroscopy or CT. The needle path was planned to avoid large nerves, vessels and pleura. All needles had a stylet and the needle diameter varied from 0.47 to 0.70 mm. The radiologist inserted the needle in three different sites of each lesion, i.e., centrally and peripherally (see Figs. 1 and 2). The needle position was checked by fluoroscopy or CT-scan before the aspiration was performed by the cytologist. The aspirates were placed onto slides, smeared and air-dried. They were routinely stained with May–Grünwald–Giemsa (MGG). In 10 cases, part of the yield was preserved for complementary studies, e.g., immunocytochemistry and karyotyping.

In 29 of the 30 patients, a FNAB from three different sites of each lesion could be done. In one

**Table 1.** *The site of the 29 lesions, tentative radiological diagnosis and final diagnosis*

| Location | (*n*) | Lodwick | Radiological diagnosis | Final diagnosis |
|---|---|---|---|---|
| Cervical vertebra | (1) | 3 | Metastasis* | Normal bone marrow |
| Thoracic vertebra | (3) | ? | Tuberculosis | Tuberculosis |
| | | 3 | Metastasis* | Myeloma |
| | | 3 | Metastasis* | Chordoma |
| Lumbar vertebra | (1) | 3 | Metastasis* | Metastasis – breast cancer |
| Pelvis | (4) | 2 | Osteomyelitis | Lymphoma |
| | | 3 | Metastasis* | Metastasis – carcinoma |
| | | 3 | Metastasis* | Myeloma |
| | | 3 | Metastasis* | Metastasis – adenocarcinoma |
| Sacrum | (2) | 3 | Post traumatic osteolysis | Normal bone marrow |
| | | 3 | Chordoma | Myeloma |
| Rib | (3) | 3 | Metastasis* | Metastasis – adenocarcinoma |
| | | 3 | Metastasis * | Lymphoma |
| | | 3 | Metastasis* | Lymphoma |
| Femur | (4) | 2 | Degenerative disease | Aseptic bone necrosis |
| | | 3 | Chondrobalstoma | GCT |
| | | 3 | MFH | MFH |
| | | 3 | Osteosarcoma | Osteosarcoma |
| Humerus | (4) | 3 | GCT | GCT |
| | | 3 | Chondrosarcoma | Chondrosarcoma grade II–III |
| | | 3 | Osteosarcoma | Osteosarcoma |
| | | 3 | Osteosarcoma | Osteosarcoma |
| Tibia | (2) | 3 | GCT | GCT |
| | | 3 | GCT | GCT |
| Fibula | (1) | 2 | Fibrous dysplasia | Fibrous dysplasia |
| Hand | (1) | - | Enchondroma | Enchondroma |
| Ulna | (1) | 2 | Osteomyelitis | Osteomyelitis |
| Clavicle | (1) | 2 | Degenerative bone cyst | Degenerative bone cyst |
| Foot | (1) | ? | Gout | Gout |
| All | (29) | | | |

*Including myeloma and lymphoma

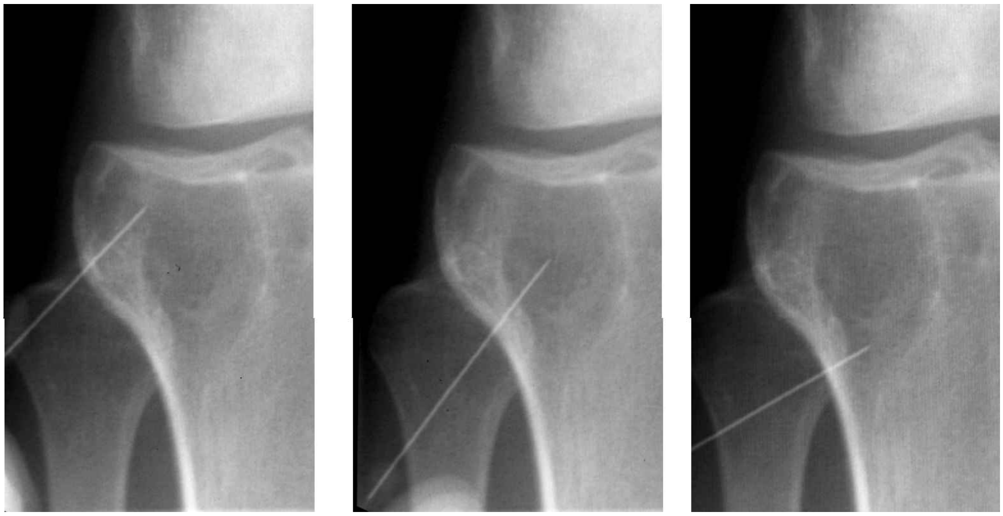

**Fig. 1.** *GCT of proximal the tibia. Fluoroscopic guidance of FNAB into three different parts of the tumour. Cranially (a), centrally (b) and caudally (c).*

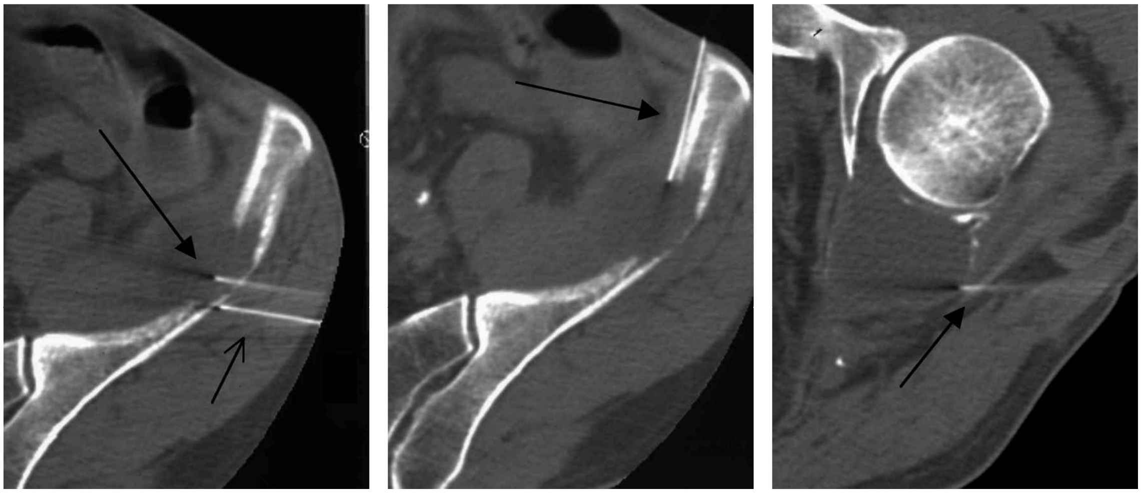

**Fig. 2.** *Plasmocytoma of the left pelvis. CT guided biopsy of three different parts of the tumour. Centrally (a), ventrally (b) and caudally (c). Biopsy needle (➡) and guiding needle (→)*

case of enchondroma of the proximal humerus, pain precluded more than one FNAB. Thus, altogether 87 aspirates were obtained from 29 patients. The site of the lesions is shown in Table 1.

### Cytology

### *Original review*

A cytologist at Karolinska Hospital reviewed the cytological slides without having any clinical or radiological information. The cytological specimens were given to the cytologist randomly. Thus, 87 specimens from 29 lesions were evaluated without knowing whether the given slides originated from the same lesion or not.

The series were cytologically grouped into four major categories: (1) non-conclusive (no or insufficient cell material), (2) benign (also inflammatory processes and normal features), (3) sarcoma, (4) other malignancy (metastasis, myeloma or lymphoma).

When all three FNABs from one lesion exhibited the same cytological picture, the case was considered cytologically homogeneous. This could also entail cases in which all aspirates were classified as non-conclusive. A case was considered cytologically heterogeneous, if there was any discrepancy between the assessments of the three FNABs. Most importantly, the assessment of the aspirates from the same case could provide one to three diagnostic alternatives. The heterogeneous group could also include cases with a mixture of conclusive and non-conclusive aspirates, albeit with only one diagnostic suggestion.

*Second opinion*

To check the results of the original reviewer the cases were also reviewed by another musculo-skeletal cytologist (Lund, University Hospital) in the same blind manner. The assessments of the two cytologists were compared and the cases were classified as compliant (no interobserver difference) or non-compliant (interobserver difference).

### Results

*Original review*

Thirteen out of the 29 cases were homogeneous, i.e., all three aspirates displayed the same cytological picture. Notably, five of these 13 cases consistently yielded non-conclusive aspirates (Table 2). Thus, 16 cases were heterogeneous, i.e., the three aspirates from the same lesion were differently classified, but only one case suggested two alternative diagnoses (Table 3; case 8 in Table 4). The remaining 15 heterogeneous cases exhibiting a mixture of conclusive and non-conclusive aspirates suggested only one diagnosis.

Altogether, 22 of the 29 cases were correctly assessed in relation to the final diagnosis. Among the seven remaining cases, there were the five consistently providing non-conclusive aspirates. Thus, two cases were incorrectly assessed. One aspirate from a GCT was classified as a sarcoma, the other two as non-conclusive. The three aspirates from another GCT were classified as benign, sarcoma and non-conclusive, respectively.

*Second opinion*

Comparison of the original and second review showed that 21 of the 29 cases were fully compliant, i.e., no interobserver difference among 63 of 87 aspirates. Closer analysis of the remaining eight discrepant cases showed that the difference pertained to 10 of 27 aspirates. In six cases, eight aspirates were classified by either of the cytologists as non-conclusive and as diagnostic by the other, but, notably, the discrepancies did not provide alternative diagnoses. However, in two cases (two aspirates), different diagnoses were suggested by the two cytologists. Thus, one or other of the two cytologists provided an incorrect diagnosis (see cases 7 and 8, Table 4).

A correct diagnosis was obtained in 20 of the 21 compliant cases. The only exception was a GCT, in which both cytologists classified one and the same aspirate erroneously as sarcoma and the two other aspirates as non-conclusive. Among the eight non-compliant cases, both cytologists provided the correct diagnosis in three (cases 1, 2 and 3, Table 4). In the remaining five cases, either of the two cytologists arrived at the correct diagnosis in three (cases 4, 5 and 6, Table 4) and at an incorrect diagnosis in another two (cases 7 and 8, Table 4).

In summary, of a total of 87 aspirates, 63 were assessed identically by the two cytologists. Altogether, they classified 39 and 42 aspirates, respectively, as non-conclusive. One single aspirate was incorrectly assessed by both cytologists and two aspirates by one or other of the two.

**Table 2.** *Original review: the 13 homogenous cases*

| Final diagnosis | *N* | Cytology |
|---|---|---|
| Five benign | 4 | Correct |
| | 1 | Non-conclusive |
| Three sarcoma | 3 | Correct |
| Five other malignancy | 1 | Correct |
| | 4 | Non-conclusive |

### Discussion

Our study of aspirates from different sites of the same bone lesion as obtained by radiologically guided FNAB suggests that the anticipated problem of bone tumour heterogeneity probably is very small. Also, the risk of incorrect diagnosis is low. The main

**Table 3.** *Original review: the 16 heterogeneous cases*

| Final diagnosis | N | Cytological aspirates 1 | 2 | 3 |
|---|---|---|---|---|
| Eight benign | 2 | Correct | Correct | Non-conclusive |
| | 4 | Correct | Non-conclusive | Non-conclusive |
| | 1 | **sarcoma*** | Non-conclusive | Non-conclusive |
| | 1 | benign** | **Other malignancy**** | Non-conclusive |
| Three sarcoma | 1 | Correct | Correct | Non-conclusive |
| | 2 | correct | Non-conclusive | Non-conclusive |
| Five other malignancy | 2 | correct | Correct | Non-conclusive |
| | 3 | correct | Non-conclusive | Non-conclusive |

*GCT
**GCT, two diagnostic alternatives. Incorrect diagnoses in bold.

**Table 4.** *The eight non-compliant cases*

| Case | Cytological aspirates | Original | Second | Final diagnosis |
|---|---|---|---|---|
| 1 | 1 | Non-conclusive | Non-conclusive | Fibrous dysplasia |
| | 2 | Benign | Benign | |
| | 3 | Benign | Non-conclusive | |
| 2 | 1 | Other malignancy | Other malignancy | Myeloma |
| | 2 | Other malignancy | Non-conclusive | |
| | 3 | Non-conclusive | Non-conclusive | |
| 3 | 1 | Sarcoma | Sarcoma | Osteosarcoma |
| | 2 | Sarcoma | Sarcoma | |
| | 3 | Sarcoma | Non-conclusive | |
| 4 | 1 | Benign | Non-conclusive | Enchondroma |
| | 2 | Non-conclusive | Non-conclusive | |
| | 3 | Non-conclusive | Non-conclusive | |
| 5 | 1 | Non-conclusive | Other malignancy | Myeloma |
| | 2 | Non-conclusive | Other malignancy | |
| | 3 | Non-conclusive | Non-conclusive | |
| 6 | 1 | Non-conclusive | Other malignancy | Cancer metastasis |
| | 2 | Non-conclusive | Non-conclusive | |
| | 3 | Non-conclusive | Other malignancy | |
| 7 | 1 | Benign | Benign | GCT |
| | 2 | Benign | Benign | |
| | 3 | Benign | **Sarcoma** | |
| 8 | 1 | Benign | Benign | GCT |
| | 2 | **Other malignancy** | Non-conclusive | |
| | 3 | Non-conclusive | Non-conclusive | |

Incorrect assessments in bold problem of FNAB pertains to the high number of non-conclusive aspirates.

It is well known that bone tumours occasionally exhibit tissue heterogeneity. However, it is questionable whether open biopsy is more reliable than FNAB in providing samples displaying these features. Although open biopsy and core biopsy entails more tissue material, it is mostly confined to a limited part of the tumour. If anything, the feasibility of obtaining cell material from different parts is increased by using FNAB (Fig. 3). Thus, the radiological guidance of the needle into multiple sites of a tumour (Figs. 1 and 2) would seem to offer an increased chance of detecting tissue heterogeneity. However, in the present limited series there was only one case per cytologist, albeit not the same (cases 7and 8, both GCT, Table 4), providing aspirates, which resulted in two diagnostic alternatives. In this context, it must be emphasised that the aspirates were given to the cytologists randomly without any clinical information, which is of decisive diagnostic importance. Notably, in the routine clinical work-up of all the 29 cases based on the same aspirates, only one diagnostic alternative was given. In the present study, heterogeneity almost exclusively pertained to the mixture of conclusive and non-conclusive aspirates from the same lesion. It

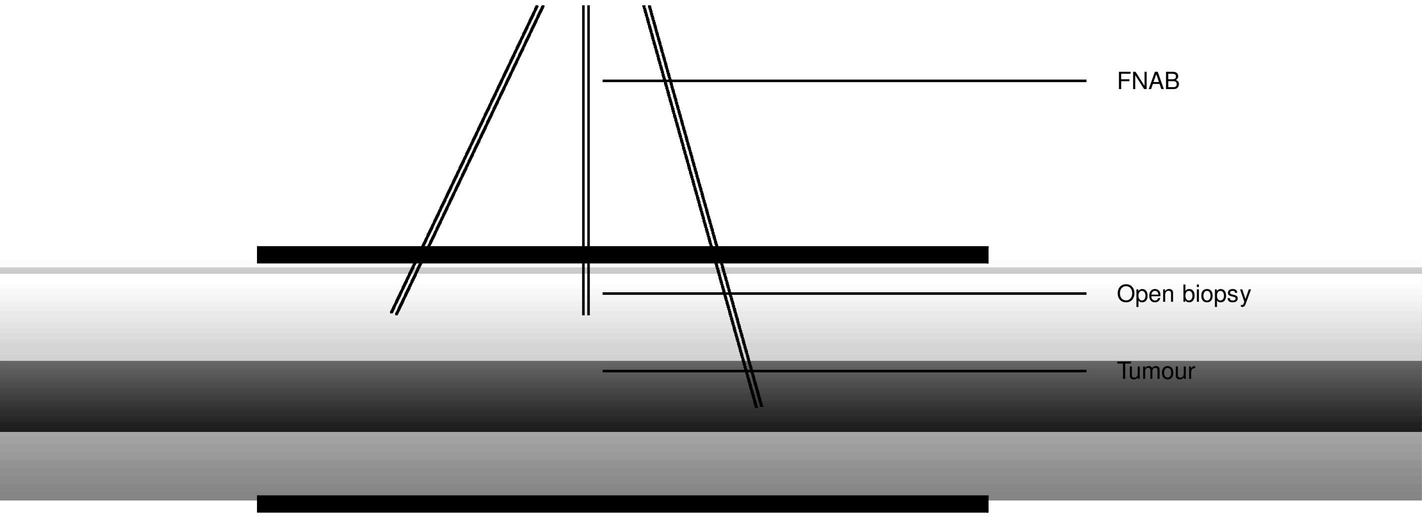

**Fig. 3.** *Open biopsy and FNAB of a bone tumour.*

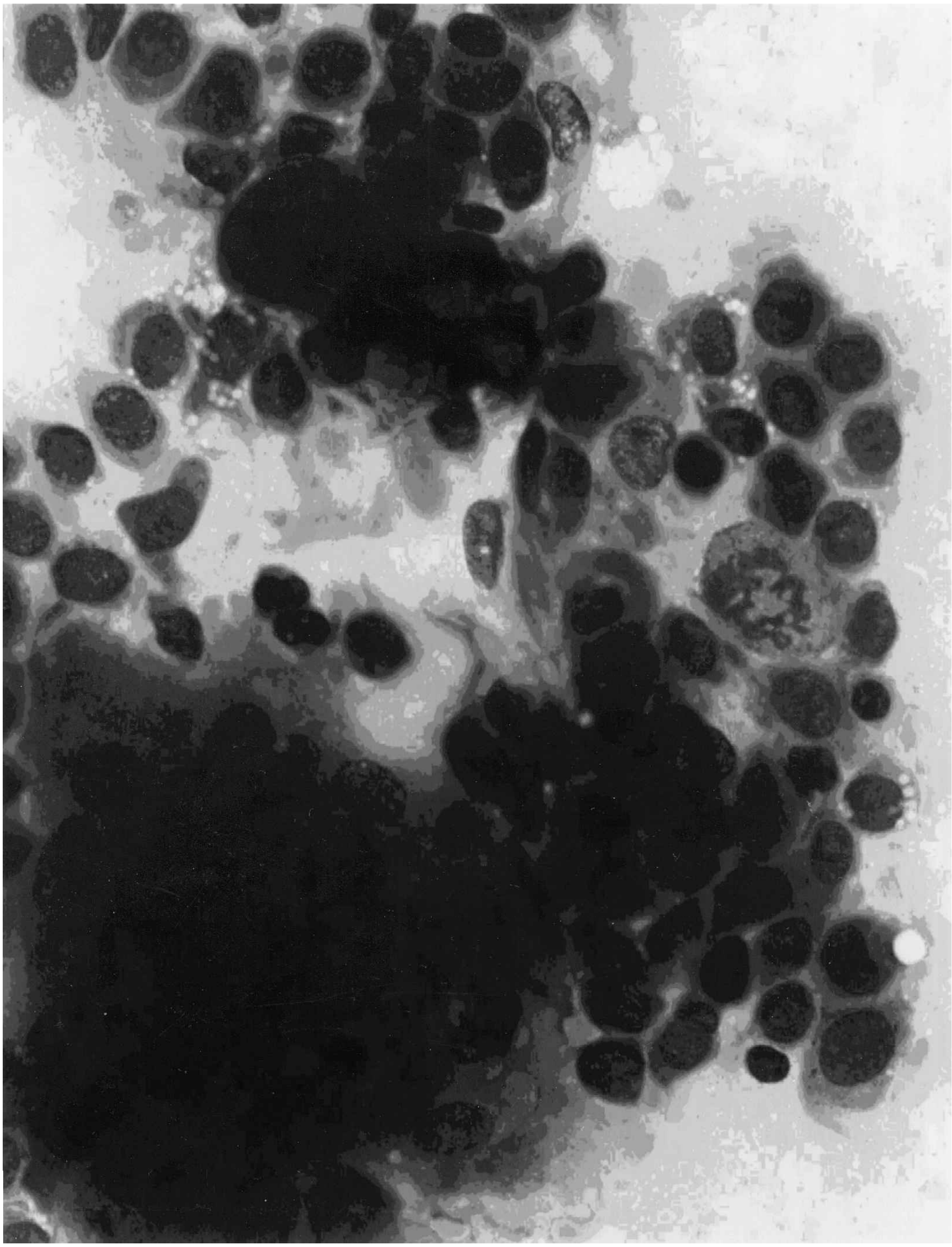

**Fig. 4.** *Cytology of GCT, with cell polymorphism and mitotic figure (MGG, x160). The aspirate was categorised as benign by one of the cytologists and as sarcoma by the other.*

remains to be established whether this should be attributed to 'true' tissue heterogeneity, sampling technique or the interpretation of cytomorphology unsupported by clinical data. In fact, the number of non-conclusive aspirates in the routine clinical work-up of the same cases was much lower. This would seem to reflect the importance of combining clinical findings and tissue morphology.

The comparison of the two cytologists' assessments showed that 63 of 87 aspirates corresponding to 21 of 29 cases were fully compliant. In six of the eight non-compliant cases, the difference between the assessments of the two cytologists pertained to eight of 18 aspirates regarded as either conclusive or non-conclusive, although no-one suggested more than one diagnosis, all correct. In the remaining two non-compliant cases only the analysis of one aspirate resulted in two different diagnoses by the two cytologists (case 7, Table 4). Another aspirate was erroneously classified by one, non-conclusive by the other. Overall, the compliance between the two cytologists should be seen as high, as only the analysis of one aspirate out of 87 resulted in two different diagnoses. Given aspirates considered conclusive, the cytomorphological assessment of cell material obtained by FNAB seems to be highly reproducible.

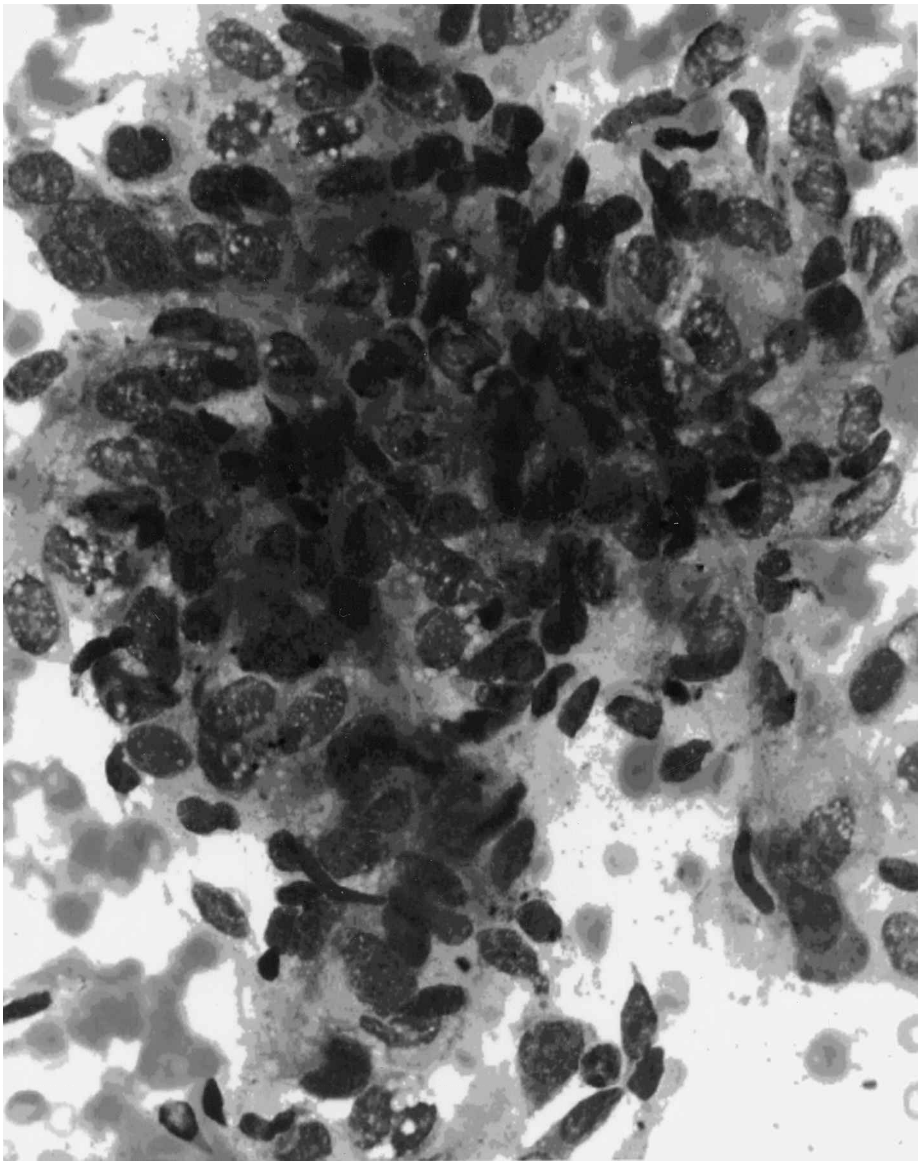

**Fig. 5.** *Cytology of GCT, shows cluster of mesenchymal cells with elongated nuclei and some vacuoles in the cytoplasm (MGG, x160). The aspirate was categorised as other malignancy by one of the cytologists and as non-conclusive by the other.*

In the present study, the diagnostic accuracy was clearly lower than that in the routine assessment of the same cases. Thus, the latter offered the correct diagnosis in 28 of 29 cases (one case non-conclusive), whereas the corresponding rate of the two blinded reviews was 22 out of 29 and 23 out of 29, respectively. This discrepancy should be attributed to the design of the study. In addition to the lack of clinical data, the reviewers were also unaware of whether or not the given aspirates originated from the same lesion. The rate of incorrect diagnosis by both of the two cytologists was two out of 29. Thus, one case of GCT was assessed by both (compliant) as a sarcoma, another two GCTs as sarcoma and other malignancy, respectively, by either of the two cytologists (Figs. 4 and 5). The failures of correctly classifying GCT may be explained by the occurrence of atypia sometimes seen in this commonly benign entity. Indeed, it is recognised that GCT, even when assessed by histopathology, can be confused with osteosarcoma and metastatic carcinoma exhibiting prominent giant cells.[8]

Given the design of the present study, the diagnostic success rate may be seen as surprisingly high. Notably, a high number of non-conclusive aspirates does not entail an increased risk of incorrect diagnosis, but rather prompts repeat FNAB or open/core biopsy. Overall, it seems that the rate of diagnostic errors by cytology of bone lesions can be kept at a low level, which seems to be comparable to histopathology provided the procedure is combined with complementary analyses like immunohistochemistry, karyotyping, etc., and, most importantly, radiological and clinical data. However, whenever cytomorphology fails to articulate with clinical findings, open or core biopsy should be considered.

### References

1. Kreicbergs A, Bauer HCF, Brosjö O, Lindholm J, Skoog L, Söderlund V. Cytological diagnosis of bone tumours. *J Bone Joint Surg [Br]* 1996; 78-Br: 258–63.
2. Wedin R, Bauer HCF, Skoog L, Söderlund V, Tani E. Cytological diagnosis of skeletal lesions. Fine needle aspiration biopsy in 110 tumours. *J Bone Joint Surg [Br]* 2000; 82-B: 673–8.
3. Söderlund V, Tani E, Skoog L, Bauer HCF, Kreicbergs A. Diagnosis of skeletal lymphoma and myeloma by radiology and fine needle aspiration cytology. *Cytopathology* 2001; 12: 157–67.
4. Huvos AG. The importance of the open surgical biopsy in the diagnosis and treatment of bone and soft-tissue tumors. *Hematol Oncol Clin North Am* 1995; 9(3): 541–4.
5. El-Khoury GA, Terepka RH, Mickelson MR, Rainville KL, Zalesli MS. Fine-needle aspiration biopsy of bone. *J Bone Joint Surg [Am]* 1983; 65-A: 522–5.
6. Bhatia A. Problems in the interpretation of bone tumors with fine needle aspiration. *Acta Cytol* 1984; 84: 1451–4.
7. Lodwick GS, Wilson AJ, Farrel C, Virtama P, Dittrich F. Determining growth rate of focal lesions of bone from radiographs. *Radiology* 1980; 134: 577–83.
8. Greenspann A, Remagen W. *Differential diagnosis of tumors and tumor-like lesions of bones and joints*. 1st edn. Philadelphia, PA: Lippincott-Raven, 1997: 319–22.
